# A Snapshot of the Prevalence and Molecular Diversity of *Legionella* *pneumophila* in the Water Systems of Israeli Hotels

**DOI:** 10.3390/pathogens9060414

**Published:** 2020-05-27

**Authors:** Eugenia Yakunin, Eszter Kostyal, Vered Agmon, Itamar Grotto, Lea Valinsky, Jacob Moran-Gilad

**Affiliations:** 1 Central Laboratories and Public Health Services, Ministry of Health, Jerusalem 9134302, Israel; eugenia.yakunin@moh.gov.il (E.Y.); vered.agmon@moh.gov.il (V.A.); itamar.grotto@moh.gov.il (I.G.); lea.valinsky@moh.gov.il (L.V.); 2 Department of Water Microbiology, Biolab Ltd., Jerusalem 9134001, Israel; ester@biolab-chemicals.com; 3 Department of Health Systems Management, School of Public Health, Faculty of Health Sciences, Ben-Gurion University of the Negev, Beer-Sheva 8410501, Israel

**Keywords:** *Legionella pneumophila*, SBT, molecular diversity, hotels

## Abstract

Exposure to *Legionella* spp. contaminated aerosols in hotel settings confers risk for travel-associated Legionnaire’s disease (TALD). In this study, we investigated the prevalence of *Legionella* contamination and its molecular diversity in hotels and resorts across Israel. The study was comprised of a convenience sample of water systems from 168 hotels and resorts countrywide, routinely inspected between March 2015 and February 2017. Isolation and quantitation of *Legionella* were performed in a water laboratory using the ISO 11731 method. The distribution of *Legionella* isolates was analyzed according to geography and source. The genetic diversity of a subset of isolates was analyzed by sequence-based typing (SBT) at the National Reference Laboratory for *Legionella* and compared to the national database. Out of 2830 samples tested, 470 (17%) obtained from 102 different premises (60% of hotels) were positive for *Legionella* spp. In 230 samples (49% of all positive, 8% of total samples), accounting for 37% of hotels, *Legionella* spp. counts exceeded the regulatory threshold of 1000 CFU/L. The most frequently contaminated water sources were cooling towers (38%), followed by faucets, hot tubs, water lines, and storage tanks (14–17% each). Furthermore, 32% and 17% of samples obtained from cooling towers and hot tubs, respectively, exceeded the regulatory thresholds. SBT was performed on 78 strains and revealed 27 different sequence types (STs), including two novel STs. The most prevalent STs found were ST1 (26%), ST87 (10%), ST93 (6%), and ST461 and ST1516 (5% each). Several *L. pneumophila* STs were found to be limited to certain geographical regions. This is the first study to investigate the prevalence and diversity of *Legionella* in hotels and resorts in Israel during non-outbreak environmental inspections. These findings will inform risk assessment, surveillance, and control measures of TALD.

## 1. Introduction

*Legionella* is a Gram-negative bacterium found ubiquitously in aqueous environments, which can multiply quickly in man-made water systems [1]. *Legionella* spp. have a complex life cycle, and exist in the environment as free-living bacteria in microbial consortia of environmental organisms or as intracellular pathogens. *L. pneumophila* has plenty of virulence factors, which it uses effectively to infect aquatic protozoa or human lung alveolar macrophages [2].

*L. pneumophila* is the major causative agent of Legionnaires’ disease (LD), a severe pneumonia with a fatality rate of up to 15%, and a flu-like illness called Pontiac fever [3,4]. Humans can contract the disease during exposure to contaminated water aerosols generated by hot and cold water systems, cooling towers, showering facilities, and spa pools [5]. *Legionella* bacteria is an opportunistic pathogen [2]. The risk factors include old age, underlying diseases, and smoking [6]. Although many *Legionella* spp. are considered potentially pathogenic for humans, *Legionella pneumophila* (Lp) causes the vast majority of LD cases, and of the 16 known Lp serogroups (sg), sg1 accounts for over 80% of LD cases [7,8]. 

Legionellosis is often associated with staying in hotel accommodations, and LD is recognized as a major form of travel-associated pneumonia (TALD) [9]. Since 2010, TALD cases have accounted for 20% of all reported LD cases in Europe each year. The number of cases reported to the European TALD surveillance scheme continues to rise annually, with a 20% increase observed between 2014 and 2015 [10]. Moreover, *Legionella pneumophila* has significant outbreak potential. Since its first fatal outbreak in a hotel in Philadelphia, United States, in 1976, many clusters and outbreaks linked to hotel settings have been investigated globally [11,12,13,14]. Factors shown to contribute to the *Legionella* spp. spread and colonization are the complexity, old age, and poor maintenance of a distribution system, warm water temperature, and the presence of amoebae [1,15,16,17]. Several recent studies have focused on the prevalence and distribution of *Legionella* in water systems of hotels in non-outbreak situations. These studies revealed variable rates of contamination and species diversity [18,19,20,21], but limited data is published on the molecular diversity of *Legionella* spp. in hotel settings [22,23,24].

In Israel, where international and domestic tourism is an important branch of the national economy, TALD has accounted for 15% of all LD cases between 2006 and 2011 [25]. According to recent national epidemiology surveillance data of the Ministry of Health, the majority of TALD cases in Israel are sporadic or imported from abroad, and no major change in trends was observed during the last decade. While isolates from TALD cases undergo molecular typing, a few of them have been linked to a specific accommodation sites. It is likely that a great proportion of cases go unnoticed, due to the mild symptoms and underdiagnosis, the long incubation period of *Legionella* spp., and the short-term nature of domestic tourism. Of note is that no comprehensive data are available concerning the abundance of *Legionella* spp. in Israeli hotel water systems. In this study, we investigated, for the first time, the prevalence and characteristics of environmental *Legionella* spp. in the Israeli hotel setting as part of routine inspections.

## 2. Results

### 2.1. Legionella Contamination Rates

**Figure 1:**
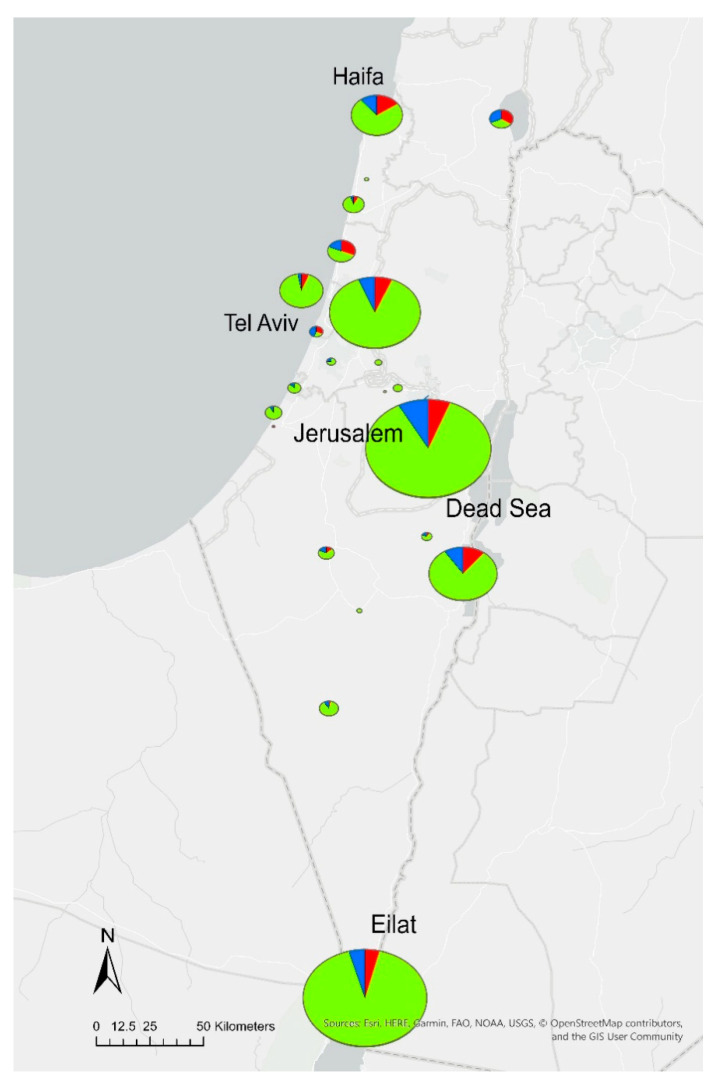
Geographic distribution and number of samples obtained from the 168 hotels and resorts included in the study. Samples are represented as pie charts at different locations; the size is proportional to the number of samples obtained from a specific location. Negative samples are shown in green, and positive and exceeding samples are shown in blue and red, respectively. Four major tourist sites with the largest number of samples (negative/positive/exceeding the regulatory thresholds) are Eilat (695/38/34), Jerusalem (662/71/52), the Tel-Aviv region (384/31/32) and the Dead Sea region (210/28/32).

During the study period, 2830 water specimens were collected routinely from the water systems of 168 hotels and resorts in six districts across Israel. *Legionella* spp. were isolated from 470 samples (17%) originating from 102 (60%) hotels. The percentage of *Legionella*-positive samples was lower in the Southern, Jerusalem, and Tel Aviv districts (13%, 15%, and 14%, respectively), of which the largest number of samples was submitted (1139, 794, and 447 samples). A higher level of contamination was found in the North (40%), but only 42 samples were collected (Table 1 and Figure 1). In 230 samples (49% of all positive, 8% of total samples), accounting for 37% of hotels, *Legionella* spp. concentrations exceeded the national regulatory thresholds. The percentage of exceeding samples per district ranged from 6% to 33% (Table 1).

Analysis of *Legionella* spp. prevalence according to sample source showed that both cold and hot water distribution systems were colonized. The leading contaminated water sources were cooling towers (38%), followed by hot tubs, outlets, and main water lines (14–17% each). Of 277 *Legionella*-positive water samples from the outlets representing hotel rooms, 166 (59.9%) and 111 (40.1%) were from hot and cold water systems, respectively. The respective positivity rates were 15.3% and 17.1%. Levels exceeding regulatory *Legionella* counts were detected in 84 samples from the outlets, of which 67 samples (79.7%) originated from hot and 17 (20.2%) from cold water systems. The respective exceedance rates above regulatory thresholds were 6.2% and 2.6%. The majority of contaminated main water lines represented hot water distribution systems and accounted for 63 (92.6%) of 68 positive samples from water lines. Only five (7.3%) positive samples were from cold water lines. Of 35 samples with levels of contamination exceeding regulatory thresholds, 33 (94.2%) were from hot water lines at a rate of 8.6%. In 74 (32%) of 232 samples from cooling towers, and 36 (17%) of 218 samples from hot tubs, levels of *Legionella* spp. exceeded the regulatory thresholds of 1000 CFU/L established for potable water and the 1 CFU/100 mL threshold for the hot tubs (Table 2).

### 2.2. Distribution of Serotypes

Serotyping of a convenience sample comprised of 162 isolates revealed that 104 isolates (64%) from 44 hotels belonged to sg2–14, and 53 (33%) isolates from 22 hotels were sg1. Both sg1 and sg2–14 were found in the water systems of nine hotels. Two percent of isolates (4/162) for which serogroup identification failed and one recognized by the kit as *Legionella* spp. were subjected to *mip* sequencing, and subsequently identified as *L*. *pneumophila* and *L. bozemanii*, respectively. Overall, Lp accounted for the vast majority of the 162 serotyped isolates (99%).

Furthermore, we analyzed serogroup prevalence according to sample type. The majority of sg2–14 isolates were recovered from hot and mixed water samples, and accounted for 81 (77.8%) of the 104 isolates. Lp sg1 isolates were predominant in cold water systems, and 40 (75.4%) of the 53 isolates originated from cold water.

### 2.3. Phylogenetic Analysis

**Figure 2:**
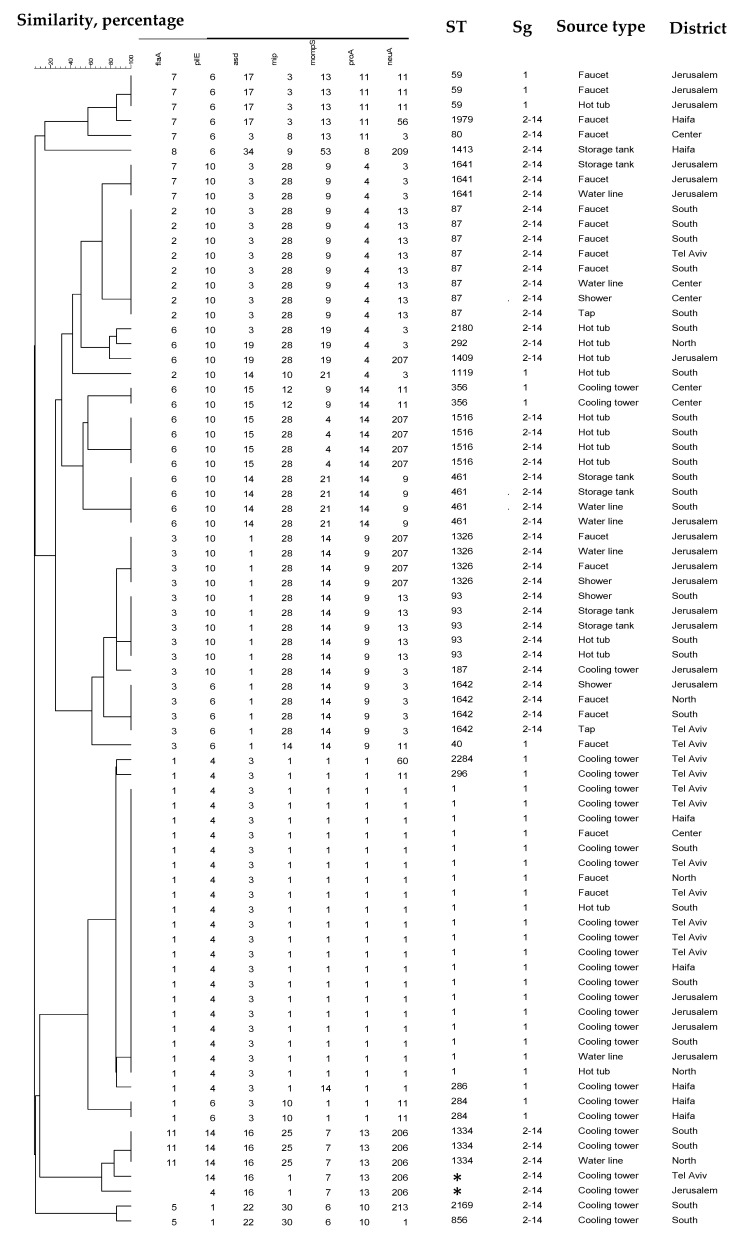
Similarity dendrogram of 78 *L. pneumophila* strains isolated from hotel and resort water systems in Israel. A phylogenetic tree was constructed using unweighted pair group method with arithmetic averages (UPGMA) clustering. The sequence type (ST), serogroup, source by category type, and region of isolation are indicated. The asterisks indicate untypeable isolates (failed *flaA* gene amplification).

Phylogenetic analysis using SBT of a subset of 78 isolates revealed 27 different STs, including two novel STs (ST2169, ST2284), with the index of diversity being 0.912. Twelve STs were associated with more than one isolate, and 16 STs were identified with one single isolate. The most prevalent STs found were ST1 (26%), ST87 (10%), ST93 (6%), and ST461 and ST1516 (5% each). Of all Lp sg1 isolates, ST1 accounted for 63% (20/32), while the leading Lp non-sg1 subtype, ST87, comprised 17% of all sg2–14 isolates (8/46). Amplification failure of the *flaA* fragment occurred in two allelic profiles (0,4,16,1,7,13,206 and 0,14,16,1,7,13,206), and therefore no STs could be obtained for them. The clustering analysis of the 27 strains is shown in Figure 2.

**Figure 3:**
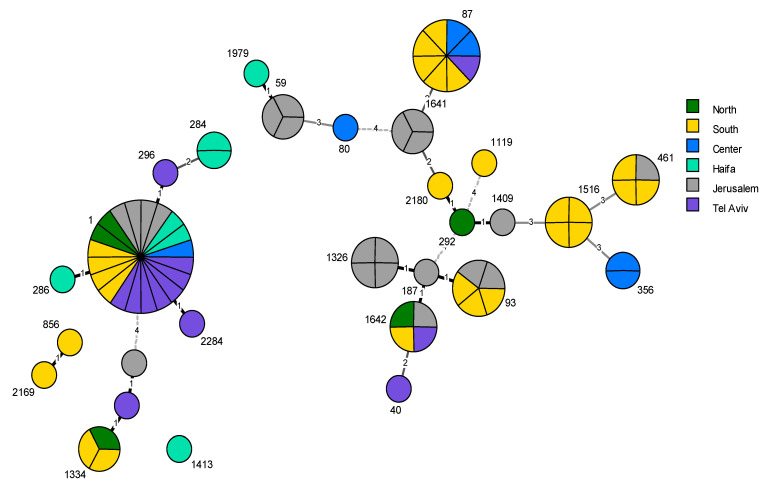
Minimum spanning tree (MST) based on sequence-based typing (SBT) profiles of 78 Lp isolates from hotel and resort water systems. Sequence type (ST) is indicated next to the circles, circle size is proportional to the number of isolates sharing the same ST, and each isolate is shown as a segment of the relevant circle. Branches connect the STs and show the genetic distance between them. STs differing by five or more alleles are not connected. Color-coding of the circles denotes geographic region. Nodes without an ST number represent strains closely related to ST1334 with failed *flaA* typing.

While several *L. pneumophila* sequence types were distributed widely throughout the country (ST1, ST1642, and ST461), a number of strains have been found to be limited to certain geographical regions. Specifically, ST59, ST1326, and ST1641 were unique to the Jerusalem district, and ST1516 was only found in the Southern region. Moreover, the Southern and Jerusalem districts displayed the most diverse *L. pneumophila* population, with 11 and 10 different STs, respectively (Figure 3). 

## 3. Discussion

The abundance of Lp in the tourism sector is a continuous focus of attention in *Legionella* research, due to its possible implications to public health. A summary of earlier publications reporting national surveys of tourist accommodations in different countries is presented in Table 3. 

This study shows, for the first time, the distribution and prevalence of *Legionella* spp. in the Israeli hotel sector. By analyzing 2830 water specimens, taken from 168 hotels over the two-year period between 2015 and 2017, we demonstrate that 60% of the examined hotels were colonized with *L. pneumophila*, and in 37% of them, the concentrations of *Legionella* in water exceeded the national regulatory thresholds. Of all 2830 specimens collected, 17% were *Legionella*-positive, with half of those exceeding threshold levels of *Legionella*.

We analyzed the results of *Legionella* quantitation, according to the category of water source, which included cooling towers, hot tubs, waterlines, showering facilities, storage tanks, and room tap water. The most affected source type was cooling towers (38%), while specimens from other sources showed lower rates of *Legionella* colonization, at around 15%. Furthermore, 32% of the samples from cooling towers exceeded the 1000 CFU/L regulatory threshold for *Legionella* concentrations, making this the water source with the highest proportion of exceeding samples.

Cooling towers are the most frequently reported water source of LD outbreaks worldwide [26,27,28], and can involve a large number of cases [29,30]. The role of cooling towers in the urban spread of Lp has also been demonstrated recently in a genomic analysis of isolates over time in Switzerland [31]. The high proportion of Lp-contaminated cooling towers reported here is a public health concern that should prompt further investigation, due to the high population density in urban areas. However, in contrast to the reports from other countries, LD cases in Israel have not been linked to cooling towers. Since not all *Legionella* spp. and Lp strains are suggested to have the capacity to cause LD [32,33], this might be a reason for the discrepancy. It would be interesting, therefore, to look specifically at the population structure of *Legionella* spp. in cooling towers nationwide.

Serotyping of a subset of 162 presumed *Legionella* isolates revealed that 33% belonged to Lp sg1, while 64% belonged to Lp sg2–14. Several studies have explored the distribution of Lp sg1 in the environment. A study from South Korea demonstrated the significant predominance of Lp sg1 in manmade water systems, including hotels, with prevalence rates up to 55% [22]. In Italy, the Lp sg1 distribution rates in the hotel setting differed greatly between two studies, at 27.7% and 55%, including mixed cultures [15,34]. On the other hand, findings from Italy [19], Greece [23], and Turkey [21] have shown that the most frequent colonizers of the hotel water systems in these studies were Lp sg2–14. 

A growing body of evidence shows the *Legionella* strains’ ability for long-term persistence in manmade water systems, without a significant fluctuation of population diversity [12,35,36]. Based on this hypothesis, we assume that our findings reflect the rates of Lp sg1 distribution in Israeli hotels, though more investigation is needed to extend our knowledge on the persistence of local Lp strains in water systems associated with different settings.

Using SBT applied on a convenience sample of 78 Lp isolates, we have identified 27 STs, including two novel STs. Nine STs belonged to sg1. Lp sg1 ST1 was the prevalent type, accounting for 26% (20/78) of the sequence-typed isolates.

ST1 has been described by numerous studies, amongst a few other STs, as a main causative agent of LD globally, supporting its high pathogenicity [37,38]. Moreover, in contrast to other highly pathogenic clinical strains rarely isolated from the environment, ST1 has been shown to be among the predominant environmental Lp sg1 strains [22,39,40,41,42,43].

In our study, ST1 comprised 63% (20/32) of all sg1 isolates from hotel water systems. The high rate of the environmental predominance of Lp sg1 ST1 corresponds with our national surveillance data, where ST1 is by far the most common cause of LD in Israel [44]. This abundance of ST1 in the environment poses a challenge for public health services, limiting their capability to ultimately identify a source of infection during investigations of ST1-associated outbreaks using traditional SBT.

Amongst the non-sg1 isolates (46/78), two isolates failed to generate a full seven-allele profile, due to no amplification of a *flaA* PCR product. Lp strains with mutations at the SBT *flaA* primer-binding site have been described elsewhere [45], including an Lp subtype from Israel (0,14,16,25,7,13,206) that had been further identified by the whole genome sequencing (WGS) approach as having the *flaA11* allele, and which has been assigned to ST1334 [46]. However, the two strains found in this study differ from ST1334 (0,4,16,1,7,13,206 and 0,14,16,1,7,13,206), supporting the idea of an ongoing dissemination of the mutation. 

Concerning the geographical distribution of the Lp population in this study, we observed a relative abundance of a number of strains in some districts in Israel. For example, sequence type ST1 was identified in each of the six Israeli districts, and ST1642 was found in four of the districts. On the contrary, other subtypes were associated with only one geographical region. For example, ST59, ST1326, and ST1641 were found in the Jerusalem district, and ST1516 was limited to the Southern region. Even though these strains are not unique to Israel, apart from ST1641, we observe their strong association with these two regions from our surveillance programs and during epidemiological investigations [47]. An explanation might be water-related differences between the regions (i.e., physical and chemical properties) caused by the climatic and topographical characteristics of the geographic regions. However, more data is needed to verify this assumption.

In our study, we have found an unexpectedly low rate of non-Lp spp. in the hotel water systems. In fact, we identified only one *L. bozemanii* strain from isolates subjected for serotyping. Other studies that have explored the environmental distribution of *Legionella* spp. have detected Lp and non-Lp co-existence in water. A recent study from the United States has demonstrated that 72 of culture-positive environmental samples collected during summer 2016, where Lp sg1 was recovered, also contained at least one other Lp or non-Lp *Legionella* spp. [30]. In another study from Crete, Greece [23], carried out from 2004 to 2011, about 50 non-Lp *Legionella* spp. were identified in the water systems of the hotel setting. Variability in the prevalence of non-Lp spp. between the studies can possibly be explained by the differences in the isolate selection procedure for subsequent analysis. In our study, the initial identification of *Legionella* spp. was carried out by serotyping, followed by the *mip* sequencing of non-groupable strains. In contrast, the application of PCR-based techniques for *Legionella* spp. screening on all samples would probably have yielded results that are more diverse. Moreover, the isolation processing methods used in our study may have reduced the detection of non-*pneumophila Legionella* spp., due to the overgrowth of other bacteria, and may have underestimated their overall abundance in samples. The membrane plating method is subject to some issues of overgrowth (especially in non-potable water), has been reinforced in a few studies [48,49], and is discussed in the new ISO 11731:2017 [50]. Thus, *Legionella* spp. distribution may not be fully represented here.

This study has several limitations. First, despite the considerable overall number of 2830 water specimens analyzed, several geographical regions, such as the Northern district, were underrepresented in our study. Second, the study was based on a convenience sample, and thus may not accurately represent the entire tourism sector in Israel, or in certain districts. 

In this survey, a large set of water samples was examined routinely without any sampling efforts, due to sporadic travel-associated LD cases or outbreaks. Therefore, our findings on *Legionella* prevalence in hotel settings in Israel are fully representative of non-outbreak-related surveillance. Regarding the molecular structure of *L. pneumophila* population, this study demonstrates, for the first time, the molecular profile of Lp strains in the water systems of Israeli hotels and resorts. 

Altogether, our findings contribute to the existing knowledge concerning the understanding of the environmental distribution of *Legionella* spp. in our region, and may facilitate international activities, such as TALD surveillance. The peculiar geographic distribution of different strains should be further investigated.

## 4. Materials and Methods

In total, 2830 convenience samples from 168 hotels and resorts were collected via routine surveillance, according to the regulations of the Israeli Ministry of Health for the prevention of *Legionella* growth in water distribution systems and hot tubs [51]. The study took place between March 2015 and the end of February 2017 across six Israeli districts (Northern, Center, Southern, Haifa, Tel Aviv, and Jerusalem). Hotels and other tourist accommodation are obliged by the regulations to monitor their water systems for the presence of *Legionella* spp. The minimum mandatory testing routine schedule depends on the hotel’s size: once every two years for sites containing <50 rooms, once a year for those with 50–300 rooms, and twice a year for those with >300 rooms. Both hot and cold water distribution systems should be tested as part of this procedure. For hot tubs, the minimum sampling routine is quarterly. Selection of the sampling points depends on a hotel water system maintenance plan and is comprised of hot and cold water from outlets representing the rooms (faucets, showers) and mains (hot water return lines, hot and cold water supply, and storage tanks): cold water from cooling towers, decorative fountains, pools, air conditioning systems, and cold/hot/mixed water from hot tubs. Samples were taken after flushing for 2 minutes and the disinfection of the outlet, as per the requirements of the Israeli Public Health guidelines for routine monitoring of water distribution systems [51]. In addition, following regulatory requirements, water systems with *Legionella* concentrations above the thresholds of 1000 CFU/L for potable water and 1 CFU/100 mL for hot tubs were re-tested after the appropriate treatment [51]. Overall, of the 168 hotel water systems included in this study, 119 were probed at least twice. At each sampling point, 1–2 water samples (hot and/or cold water) were collected in 1 L sterile plastic bottles containing sodium thiosulfate, in order to neutralize the residual-free chlorine. All water samples were stored at 4 °C and processed within 24 h of their collection. 

The detection and quantitation of *Legionella* spp. were performed in a certified water testing laboratory per the ISO 11731-2:2004 method [52]. Potable and non-potable water samples were filtered with 0.45 µm sterile gray membrane filter paper, treated with 30 mL of acid buffer containing 0.2 M KCL and 0.2 M HCL for 5 min, and washed with 20 mL of PAGE’s saline. Water samples originated from cooling towers and fountains were processed in four dilutions (1:10000, 1:1000, 1:100, and 1:10), in order to avoid the overgrowth of microbial flora. Membranes were transferred to Glycine Vancomycin Polymyxin Cycloheximide (GVPC) medium (cat. no. 257007, BD, Heidelberg, Germany) and after incubation at 35 ± 0.5 °C for 10 days, colonies suggestive of *Legionella* spp. were subcultured to Buffered Charcoal Yeast Extract (BCYE) and 5% sheep blood agar media (P073 and P049, HyLabs, Rehovot, Israel). Subsets of representative isolates identified as *Legionella* spp. were regularly referred to the National Reference Laboratory for *Legionella* at the Ministry of Health, according to regulations [51].

The total amount of 164 *Legionella* isolates from hotel water systems was obtained during the two-year study. Serotyping was performed with the Legionella Latex Test kit (Cat. No. DR0800, Oxoid, Basingstoke, UK). 

Strains not readily confirmed by serotyping as *L. pneumophila* were identified to species level by sequencing the *mip* gene, as described by Ratcliff et al. [7], and comparing the sequence to the *mip* database [53]. The molecular characterization of *L. pneumophila* strains was conducted according to the European Society of Clinical Microbiology and Infectious Diseases (ESCMID) Study Group for *Legionella* Infections (ESGLI) sequence-based typing (SBT) scheme [54,55]. The choice of the isolates subjected to SBT monthly was based on the data provided by the referring laboratory and guided by epidemiological and risk assessment criteria, such as high *Legionella* CFU counts, a source type with high public health risk potential, or a new sampling site. After the exclusion of duplicate isolates arising from the same sampling points, 78 isolates were examined in this study. Sequences obtained by Sanger sequencing were analyzed with the BioNumerics software (Version 7.6, Applied Maths) and compared to the ESGLI database for assigning the ST. New allelic profiles were submitted to the ESGLI SBT database [56]. The strain diversity index was calculated according to the modified method of Hunter and Gaston [57].

BioNumerics software (Version 7.6, Applied Maths) was used for phylogenetic analysis. Clustering was created using the unweighted pair group method with arithmetic averages (UPGMA) [58]. The minimum spanning tree (MST) was created using a predefined MST for the categorical data template, with single- and double-locus variance priority rules. Geomap was created using ArcGIS Pro 2.5 (Esri, Redlands, CA, USA).

## Figures and Tables

**Table 1:** Distribution of premises and samples, according to administrative region.

District	Total Tested		Positive Samples		Exceeding Samples ^1^	
	No. of Hotels	No. of Samples	No. of Hotels	No. of Samples (% Per District)	No. of Hotels	No. of Samples (% Per District)
North	3	42	2	27 (64)	1	14 (33)
Center	9	207	6	54 (26)	4	30 (14)
South	78	1139	44	151 (13)	28	72 (6)
Haifa	9	201	7	52 (26)	4	30 (15)
Tel Aviv	20	447	12	63 (14)	8	32 (7)
Jerusalem	49	794	30	123 (15)	17	52 (7)
**Total**	**168 ^a^**	**2830**	**101 ^b^**	**470 (17)**	**62 ^c^**	**230 (8)**

^1^ Cut-off values of 1000 CFU/L for all categories and 1 CFU/100 mL for hot tubs, according to the national regulations; ^a^ Out of 168 hotels and resorts tested for *Legionella* spp., 119 were tested more than once (range of 1–25 sampling days, median 2); ^b^ Of 101 hotels with positive samples under the regulatory cutoffs, 56 were sampled at least twice (range of 1–17 sampling days, median 2); ^c^ Of 62 hotels with *Legionella* concentrations above the national regulatory cutoffs, 29 were sampled at least twice (range of 1–12 sampling days, median 1).

**Table 2:** Prevalence of *L. pneumophila*, according to source type.

Source Type	No. of Samples				Positive Samples		Exceeding Samples ^1^	
	Cold Water	Hot Water	Mixed Water ^2^	Total	No. of Samples	Per Category (%)	No. of Samples	Per Category (%)
Outlet	649	1084		1733	277	16%	84	5%
Main water line	96	383		479	68	14%	35	7%
Cooling tower	232			232	87	38%	74	32%
Hot tub	5	9	204	218	36	17%	36	17%
Fountain	24	4		28	1	4%	0	0%
Pool	11			11	0	0%	0	0%
Air conditioning	4			4	1	25%	1	25%
Not available ^3^	38	87		125	0	0%	0	0%
**Total:**	**1059**	**1567**	**204**	**2830**	**470**	**17%**	**230**	**8%**

^1^ Cut off values of 1000 CFU/L for all categories and 1 CFU/100 mL for hot tubs, according to the national regulations; ^2^ According to the national regulations, the water temperature range of 37–39 °C for hot tubs can be achieved by mixing hot and cold water. ^3^ Source type not indicated in the laboratory requisition form accompanying the samples.

**Table 3:** Summary of earlier publications reporting national surveys of tourist accommodations in different countries.

Author	Country of Study	Year of Publication	Geography	Sample Selection	Sample Size	Time of Study	Laboratory Methods
Borella et al.	Italy	2005	Five representative cities, northern, central, and southern Italian regions	The hotels were selected based on the water distribution systems in the cities, the characteristics of the buildings, and hotel cooperation.	119 water samples from 40 hotels (3–5 samples from each hotel)	September 2003–July 2004	*Legionella* isolation, enumeration and serotyping; PFGE analysis; physical and chemical water analyses
Lee et al.	South Korea	2010	Seven geographic regions throughout South Korea	The number of samples and isolates depended on the number of facilities located in each region	4938 water samples from water systems of different settings, including hotels.	June–September 2008	*Legionella* isolation, enumeration and serotyping; molecular identification of *L*. spp (16S rRNA, *mip*, or *rpoB*); SBT
Napoli et al.	Italy	2010	Southeastern Italy	Representative samples from different building types and water systems. Re-inspection samples excluded.	13,286 water samples, including 5009 samples from 305 hotels	January 2000–December 2009	*Legionella* isolation, enumeration, and serotyping
Bonetta et al.	Italy	2010	Northern, central, and southern Italy	Samples representative of 18 towns and types of water systems.	76 water samples from 19 hotels	October 2006–February 2007	*Legionella* isolation, enumeration, and serotyping; real-time PCR; physical and chemical analyses
Chochlakis et al.	Greece	2013	Four regions of Crete island	Eight to 15 representative samples from each hotel, depending on hotel size and water system type.	1494 water samples from 124 hotels	2004–2011	*Legionella* isolation, enumeration, and serotyping; molecular identification of *Legionella* spp (16S rRNA, *mip*); MALDI-TOF mass spectrometry; SBT;
Sepin Özen et al.	Turkey	2017	Antalya region	Samples from different water systems	1403 water samples from 54 hotels	January–December 2010	*Legionella* isolation, enumeration, and serotyping
